# Infective endocarditis with *Lactococcus garvieae *in Japan: a case report

**DOI:** 10.1186/1752-1947-5-356

**Published:** 2011-08-09

**Authors:** Yukiko Watanabe, Toshio Naito, Ken Kikuchi, Yu Amari, Yuki Uehara, Hiroshi Isonuma, Teruhiko Hisaoka, Terutoyo Yoshida, Kenji Yaginuma, Norihide Takaya, Hiroyuki Daida, Keiichi Hiramatsu

**Affiliations:** 1 Department of General Medicine, Juntendo University School of Medicine, 2-1-1 Bunkyo-ku, Hongo, Tokyo 113-8421, Japan; 2 Department of Infection Control Science, Juntendo University School of Medicine, 2-1-1 Bunkyo-ku, Hongo, Tokyo 113-8421, Japan; 3 Department of Cardiology, Juntendo University School of Medicine, 2-1-1 Bunkyo-ku, Hongo, Tokyo 113-8421, Japan; 4 Department of Fisheries, Faculty of Agriculture, Miyazaki University, Miyazaki City, Miyazaki 889-2192, Japan

## Abstract

**Introduction:**

*Lactococcus garvieae *is a well-recognized fish pathogen, and it is considered a rare pathogen with low virulence in human infection. We describe the 11th case of *L. garvieae *infective endocarditis reported in the literature, and the first reported case in Japan.

**Case presentation:**

We report a case of a 55-year-old Japanese woman who had native valve endocarditis with *L. garvieae*. The case was complicated by renal infarction, cerebral infarction, and mycotic aneurysms. After anti-microbial treatment, she was discharged from the hospital and is now well while being monitored in the out-patient clinic.

**Conclusion:**

We encountered a case of *L. garvieae *endocarditis that occurred in a native valve of a healthy woman. The 16S ribosomal RNA gene sequencing was useful for the identification of this pathogen. Although infective endocarditis with *L. garvieae *is uncommon, it is possible to treat high virulence clinically.

## Introduction

*Lactococcus garvieae *was first isolated from cases of bovine mastitis [1], and it is rarely reported in human infection. The first human infection was documented as infective endocarditis (IE) in 1991 [2]. Since then, it has been identified as a pathogen in IE [3-9], liver abscess [10], and osteomyelitis [4]. We describe the 11th case of *L. garvieae *IE reported in the literature, and the first reported case in Japan.

## Case presentation

A 55-year-old healthy Japanese woman presented to our hospital with a two-month history of malaise and myalgia, and she had had a fever for one month. The patient had no significant medical history and no relevant history regarding pets, travel, dental treatment, or drug abuse. Her occupational history was that she worked at an import grocery store. During her physical examination, we noted her body temperature of 37.6°C, a grade 2 systolic murmur at the apex, and a painful 1 mm black induration on her right forefinger. Trans-thoracic echocardiography showed a 1 cm mobile mass on the mitral valve with moderate mitral regurgitation. Her laboratory tests showed a white blood cell count of 8200/μL, a C-reactive protein level of 6.6 mg/dL, and a hemoglobin level of 9.9 g/dL. Gram-positive cocci forming pairs or short chains were shown by Gram staining of three sets of blood cultures drawn at the time of admission.

IE was diagnosed on the basis of the Duke criteria for the diagnosis of infective endocarditis [11] (two major and two minor criteria) and antibiotic treatment with benzylpenicillin 3 million units every four hours and gentamicin 60 mg every eight hours was started, as *Streptococcus *sp. was suspected as the causative pathogen. Subsequently, the rapid ID32 Strep (bioMérieux, Marcy-l'Etoile, France) profile (code 34323501010) revealed *L. garvieae*. We also performed molecular species identification by 16S ribosomal RNA gene (16S rRNA) sequencing, and the almost full-length 16S rDNA matched *L. garvieae *(99.9%). The results of an anti-microbial susceptibility test using Etest strips (AB Biodisk, Dalvagen, Solna, Sweden) showed the following values: erythromycin 0.25 mg/L, clindamycin ≥ 256 mg/L, vancomycin 0.38 mg/L, linezolid 2.0 mg/L, penicillin 0.5 mg/L, ceftriaxone 0.38 mg/L, gentamicin 1.5 mg/L, and streptomycin 64 mg/L. Considering these results, we changed the anti-microbial treatment to intravenous ceftriaxone 2 g every 12 hours and gentamicin 80 mg every eight hours intraveneously.

**Figure 1:**
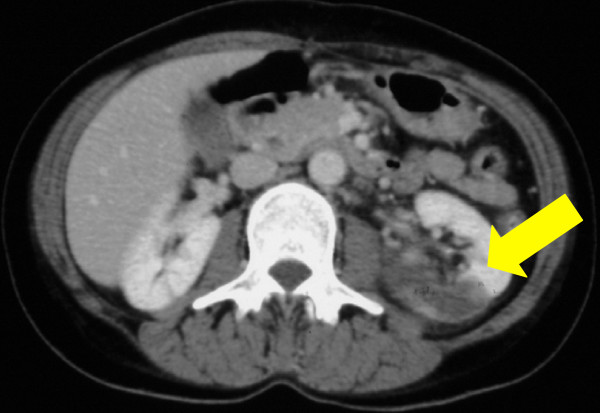
**Contrast-enhanced abdominal computed tomography**. Yellow arrow indicates left renal infarction.

**Figure 2:**
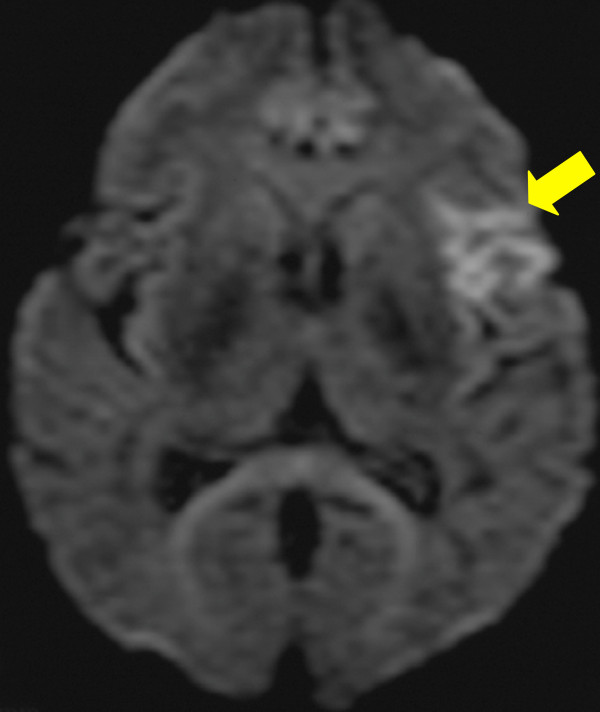
**Diffusion-weighted MRI scan**. Yellow arrow indicates the middle cerebral artery areas of high signal intensity.

**Figure 3:**
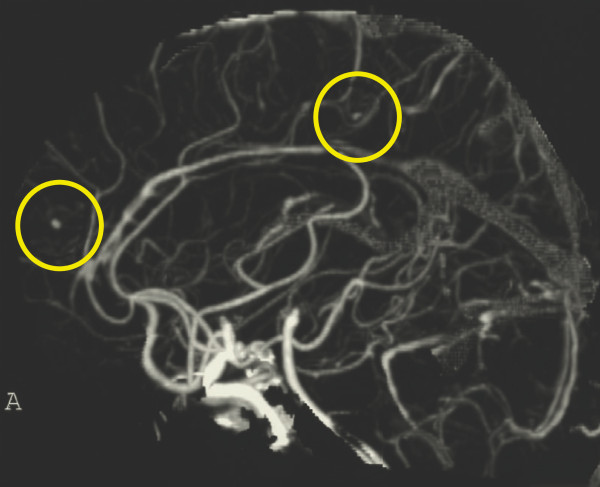
**Three-dimensional computed tomography angiography**. The two yellow circles indicate cerebral mycotic aneurysms.

Meanwhile, her body temperature rose to 39°C with left lower back pain on her sixth day in the hospital. Left-sided renal infarction was diagnosed on the basis of contrast-enhanced computed tomography (CT) (Figure 1). On the 12th hospital day, cerebral infarction occurred, followed by a sudden onset of right-sided hemiparesis and slurred speech (National Institutes of Health Stroke Scale (NIHSS) score 12). Diffusion-weighted MRI showed a cerebral infarction of the middle cerebral artery area (Figure 2). Furthermore, cerebral mycotic aneurysms were detected by three-dimensional CT angiography on the 24th hospital day (Figure 3). The patient also developed aspiration pneumonia after the cerebral infarction complication.

Anti-microbial treatment was completed in nine weeks, including the treatment for pneumonia. Several repeat blood cultures thereafter were all negative. The vegetation on the mitral valve was no longer detected by trans-esophageal echocardiography performed just before she was discharged from the hospital. We found by CT that her left-sided renal infarction had improved, and the consequences of her cerebral infarction had also improved with only right-sided muscular weakness (NIHSS score 2). She was discharged from the hospital, and she is now well while being monitored in the out-patient clinic.

## Discussion

Genus *Lactococcus *was separated from genus *Streptococcus *in 1985 based on DNA-DNA relatedness and 16S rRNA sequencing data published by Ludwig *et al. *[12]. The genus *Lactococcus *was classified into eight species and sub-species by Facklam and Elliott in 1995 [13] and Pu *et al. *in 2002 [14]: *L. lactis *ssp. *lactis, L. lactis *ssp. *cremoris, L. lactis *ssp. *hordniae, L. garvieae, L. piscium, L. plantarum, L. raffinolactis*, and *L. xyloses*. Cases involving the *L. garvieae *pathogen in human infection are rarely reported [2-10]. The major clinical presentation is IE, which has been reported in 10 cases in the literature [2-9]. Five of ten cases involved native valves [3,6-9], and four cases required surgery for valve replacement [3,6,8,9]. In addition, two cases were complicated by cerebral infarction or hemorrhage [3,9]. *L. garvieae *IE is uncommon; however, the virulence of *L. garvieae *might be underestimated. The treatment for *L. garvieae *IE is not standardized, because the exact criterion of the susceptibility test has not been established. In the published cases, penicillin combined with gentamicin, amoxicillin combined with netilmicin, ampicillin, ceftriaxone, and vancomycin were used as anti-microbial treatment. In our case, the results of the anti-microbial susceptibility test of the isolate using the Etest method was susceptibility to ceftriaxone (0.38 mg/L), erythromycin (0.25 mg/L), vancomycin (0.38 mg/L), and linezolid (2 mg/L), less susceptibility to penicillin (0.5 mg/L), and resistance to clindamycin (> 256 mg/L).

Elliott and Facklam [15] reported that clindamycin resistance is a feature of *L. garvieae*, and this feature can be used to distinguish it from other lactococci. To confirm the precise identification, various molecular techniques are available: whole-cell protein analysis, 16S rRNA gene sequencing, and *sodA*_int _(an internal fragment of the *sodA *gene encoding the manganese-dependent superoxide dismutase) gene sequencing.

*L. garvieae *has pathogenicity for several fish species, ruminants, and humans. Therefore, it could be a potentially zoonotic infection. Investigators in one recent report hypothesized that eating infected raw fish carrying *L. garvieae *caused its infection [7]. To investigate the relationship between our isolate and fish isolates, the immunological property was examined by using several anti-sera against *L. garvieae *that have previously been produced by fish in Japan. The results showed that our isolate did not have a capsule detected by any anti-capsule antibody for fish isolates. Pulsed-field gel electrophoresis also showed that the pattern of our isolate was quite different from that of fish-derived strains (data not shown). Vela *et al. *[16] found great phenotypic heterogeneity and genetic diversity among the *L. garvieae *isolates from fish, cows, water buffalo, and humans, with a generally good correlation between the phenotypic and genetic properties of *L. garvieae*. As the origin of the L. garvieae strain in our patient could not be clarified by the investigations used to detect Japanese fish pathogens, other causes of her infection, such as cow's milk or cheeses, are possible. To confirm the etiology of the transmission of L. garvieae, larger molecular epidemiological studies including investigating causes from the environment, animals, and humans must be carried out.

## Conclusion

Herein we report the first case of *L. garvieae *IE in Japan. Molecular biological investigation techniques such as 16S ribosomal RNA gene sequencing are useful for the identification of *L. garvieae*. Although *L. garvieae *is considered a rare pathogen with low virulence in human infection, it could cause native valve endocarditis in healthy people. It might be necessary to review the precise estimation of the pathogenicity of *L. garvieae*.

## Consent

Written informed consent was obtained from the patient for publication of this case report and any accompanying images. A copy of the written consent is available for review by the Editor-in-Chief of this journal.

## Competing interests

The authors declare that they have no competing interests.

## Authors' contributions

YW drafted and edited the manuscript. TN, KK, YA, YU, HI, TH, and KH helped draft the manuscript. KY, NT, and HD helped in the patient's treatment after the diagnosis was made. TY helped in the investigation of *L. garvieae *as a fish pathogen. All authors read and approved the final manuscript.
